# Refining the serum miR-371a-3p test for viable germ cell tumor detection: identification and definition of an indeterminate range

**DOI:** 10.21203/rs.3.rs-2644890/v1

**Published:** 2023-03-21

**Authors:** John Lafin, Cinzia Scarpini, Armon Amini, Bendu Konneh, Jeffrey Howard, Thomas Gerald, Michelle Nuno, Jin Piao, Anna Savelyeva, Zhaohui Wang, Jeffrey Gagan, Liwei Jia, Cheryl Lewis, Sarah Murray, Yun Sawa, Vitaly Margulis, Solomon Woldu, Douglas Strand, Nicholas Coleman, James Amatruda, Lindsay Frazier, Matthew Murray, Aditya Bagrodia

**Affiliations:** The University of Texas Southwestern Medical Center; University of Cambridge; The University of Texas Southwestern Medical Center; The University of Texas Southwestern Medical Center; The University of Texas Southwestern Medical Center; The University of Texas Southwestern Medical Center; University of Southern California; University of Southern California; The University of Texas Southwestern Medical Center; The University of Texas Southwestern Medical Center; The University of Texas Southwestern Medical Center; The University of Texas Southwestern Medical Center; The University of Texas Southwestern Medical Center; University of California, San Diego; University of California, San Diego; The University of Texas Southwestern Medical Center; The University of Texas Southwestern Medical Center; The University of Texas Southwestern Medical Center; University of Cambridge; University of Southern California; Dana-Farber/Boston Children's Cancer and Blood Disorders Center; University of Cambridge; University of California, San Diego

**Keywords:** Germ cell tumor, MicroRNA, Retroperitoneal lymph node dissection, Serum biomarker

## Abstract

Circulating miR-371a-3p has excellent performance in the detection of viable (non-teratoma) GCT pre-orchiectomy; however, its ability to detect occult disease is understudied. To refine the serum miR-371a-3p assay in the minimal residual disease setting we compared performance of raw (Cq) and normalized (ΔCq, RQ) values from prior assays, and validated interlaboratory concordance by aliquot swapping. Revised assay performance was determined in a cohort of 32 patients suspected of occult retroperitoneal disease.

Assay superiority was determined by comparing resulting receiver-operator characteristic (ROC) curves using the Delong method. Pairwise t-tests were used to test for interlaboratory concordance. Performance was comparable when thresholding based on raw Cq *vs*. normalized values. Interlaboratory concordance of miR-371a-3p was high, but reference genes miR-30b-5p and cel-miR-39-3p were discordant. Introduction of an indeterminate range of Cq 28–35 with a repeat run for any indeterminate improved assay accuracy from 0.84 to 0.92 in a group of patients suspected of occult GCT. We recommend that serum miR-371a-3p test protocols are updated to a) utilize threshold-based approaches using raw Cq values, b) continue to include an endogenous (e.g., miR-30b-5p) and exogenous non-human spike-in (e.g., cel-miR-39-3p) microRNA for quality control, and c) to re-run any sample with an indeterminate result.

## Introduction

Correct staging in early stage germ cell tumor (GCT) patients is critical for identifying patients best served with surveillance versus primary management with retroperitoneal lymph node dissection (RPLND), chemotherapy, or radiotherapy [1, 2]. In patients with clinical stage I (CS I) GCT, up to 97% of seminomas and 60% of non-seminomas recur on surveillance without marker elevation [3-5]. Additionally, 26% of patients with negative STM and cross-sectional imaging undergoing RPLND are found to have viable tumor [6]. Consequently, the performance characteristics of current STM introduces substantial risk of under- and over-treatment.

The superior performance of circulating microRNAs (miRNAs), particularly miR-371a-3p, to detect GCT is well documented. An agreed, protocolized standard for definition of positive and negative miR-371a-3p results is lacking. The absence of a standard protocol in combination with the inherent sensitivity of the test has contributed to interlaboratory heterogeneity, making comparisons difficult and limiting widespread clinical adoption [7].

We address these issues by performing interlaboratory sample exchange experiments and re-evaluating analytic pipelines for calling results. In addition to positive and negative calls, we identify an indeterminate range, which we then validate in an independent patient cohort undergoing primary RPLND. These changes improve assay performance, particularly specificity and negative predictive value (NPV), which upon clinical implementation will reduce potential over-treatment of patients without true minimal residual disease.

## Methods

### Patient Population

Thirty-two chemotherapy-naïve patients underwent primary RPLND for clinical stage I or II GCT. Serum was obtained immediately prior to RPLND. Bilateral full-template or extended modified template nerve-sparing RPLND was per surgeon discretion. Baseline clinicopathologic data were collected (Table 1). Samples were classified as either ‘Control’ (pure teratoma or no GCT), or ‘Viable GCT’ [seminoma or nonseminomatous GCT (NSGCT)].

All experimental protocols were approved by an Institutional Review Board at The University of Texas Southwestern Medical Center (STU 102010-051). Informed consent was obtained from all subjects and/or their legal guardians prior to their inclusion in the study. The authors confirm that all methods described in this manuscript were performed in accordance with the relevant guidelines and regulations.

### MiRNA isolation and quantification

RNA extraction and serum miRNA quantification were performed as described [8]. Primers and probes used are detailed in **Supplementary Table 1.** To calculate relative quantification (RQ), the ΔΔCq method was used, with the mean of four normal control human male serum samples (males between age 18–45 years) used as reference.

### Concordance studies

Serum aliquots were shipped between the two research laboratories of Cambridge, UK and University of Texas Southwestern, US priority overnight on dry ice. Upon receipt, sample inspection confirmed that none had thawed. Each site followed an identical protocol to yield raw Cq and normalized (ΔCq and RQ) values, which were then compared against one another.

### Cq *vs*. RQ performance

Raw (Cq values) and normalized (ΔCq and RQ values) data from two studies previously published from our group were utilized [9, 10]. Optimal thresholds were calculated for each metric using the Youden index [11] and sensitivity, specificity, and area under the receiver-operating characteristic curve (AUC) were calculated.

### Establishment and assessment of an indeterminate range

All runs included in our two previous reports [9, 10], including any technical replicate PCR runs undertaken, were pooled and grouped based on histology (Control or Viable GCT). An indeterminate range was defined as the 95% confidence interval of the distribution of the first (lower Cq, higher apparent abundance) raw Cq peak, rounded to whole numbers (down at the lower bound and up at the upper bound) and subsequently formally assessed for change in assay performance.

### Statistical analysis

Statistical significance for intergroup differences of clinicopathologic data was determined using the Kruskal-Wallis test with Dunn’s *post-hoc* test. Concordance was assessed by a pairwise t-test. Performance characteristics, including sensitivity, specificity, NPV, positive predictive value (PPV), accuracy, and AUC were calculated using *R* version 4.1.2 with the pROC package (version 1.18.0) and tidyverse metapackage (version 1.3.1) [12-14]. AUC values were compared using the roc.test function in pROC with default parameters. Two-tailed *p* < 0.05 was statistically significant.

## Results

Thresholding on Cq simplifies the serum miR-371a-3p test without affecting assay performance

The requirement for a normal control serum sample in each assay run for normalization is costly and adds another potential source of variation. To determine if assay normalization is required, we examined our previously published data from samples taken pre-orchiectomy [10] and pre-RPLND [9]. We examined four metrics with varying levels of normalization- Cq (raw value), ΔCq (Cq normalized to internal control miR-30b-5p), corrected ΔCq (ΔCq corrected with an external control cel-miR-39-3p), and RQ (corrected ΔCq of sample normalized to corrected ΔCq of normal serum).

Calculated sensitivity and specificity were both greater than 0.9 in all cases and did not change appreciably across any of the metrics tested, Table 1. AUC was 0.97–0.99 for all four metrics, and none were statistically different from one another (all *p* > 0.05). These results suggest that normalization to endogenous or exogenous controls, or normal healthy serum, does not impact the performance of the serum miR-371a-3p assay.

**Figure 1:**
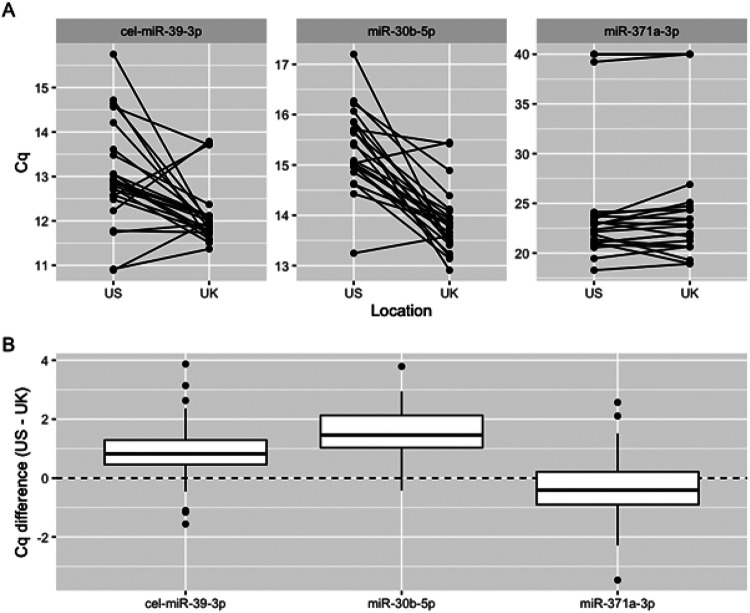
Raw miR-371a-3p Cq is concordant across laboratory sites. **A:** Comparison of raw Cq values for cel-miR-39-3p (external spike-in control), miR-30b-5p (internal reference gene), and miR-371a-3p at the US and UK sites. **B:** Boxplot of Cq differences (US – UK).

To examine interlaboratory variation, we conducted a concordance study between the two laboratories. Aliquots of the same serum sample collection were exchanged, and both sites ran identical protocols. miR-371a-3p Cq was highly concordant, with a mean difference of < 0.5 cycles between sites (*p* = 0.251), Fig. 1. The exogenous non-human spike-in control cel-miR-39-3p was discordant (*p* = 0.002), likely due to separate preparations of highly concentrated standards. Surprisingly, the endogenous control, miR-30b-5p, was also discordant (*p* < 0.001). These results suggest that this normalization process introduces additional variation and contributes to interlaboratory heterogeneity. We therefore recommend use of raw Cq values for cutoffs for the serum miR-371a-3p test going forwards.

Identification and establishment of an indeterminate range

The serum miR-371a-3p test is extremely sensitive, due in part to the pre-amplification step used prior to qPCR, which also exposes to risk of false positives. This risk is already heightened by the need to open PCR tubes following pre-amplification to set-up the qPCR, which may inadvertently spread amplification products. The inclusion of a water (‘no template’) control (NTC) sample initiated at the reverse transcription step is recommended to combat this— a positive qPCR result on NTC suggests such upstream contamination. However, we noted occasional cases where known control samples would yield an inconsistent/stochastic positive result despite a negative NTC sample result on the same qPCR run. Repeating these samples from the reverse transcription step usually yielded the anticipated negative result. In contrast, repeating runs on samples from patients with pathologically verified disease typically returned similar Cq values. Examples of repeated runs for pathologic negative and positive samples are presented in **Supplementary Fig. 1**.

To investigate the above observation, we aggregated a total of 150 runs from our previously published studies [9, 10]. We examined the distribution of Cq values split by group, Control *vs*. Viable GCT, **Supplementary Fig. 2A**. Individual sample Cq values are displayed in **Supplementary Fig. 2B**. The samples in the Viable GCT group show a broad distribution with a mean Cq and standard deviation (SD) of 26.4 ± 4.33. This wide distribution is expected given the heterogenous population with differing amounts of disease burden. However, the distribution of Cq values in the Control group appeared to be bimodal, with the mean Cq of the first peak at 32.2 ± 1.53, and the mean Cq of the second peak at 39.8 ± 0.7. The mean of the second peak is anticipated, as undetected samples are assigned Cq of 40. We were surprised that approximately 25% of all runs in the Control group fell into the first peak. Two separate research laboratories (Cambridge, UK; UTSW, Dallas, US) and one clinical laboratory (Department of Pathology, UTSW, Dallas, US) all independently reported this observation, indicating that this is unlikely to be due to technical errors. We have not found any reliable predictor for this assay behavior; it appears to be an entirely stochastic and non-predictable event. This suggests that as currently applied, the qPCR-based serum miR-371a-3p assay has an approximately 25% chance to misclassify any true negative as positive.

**Figure 2:**
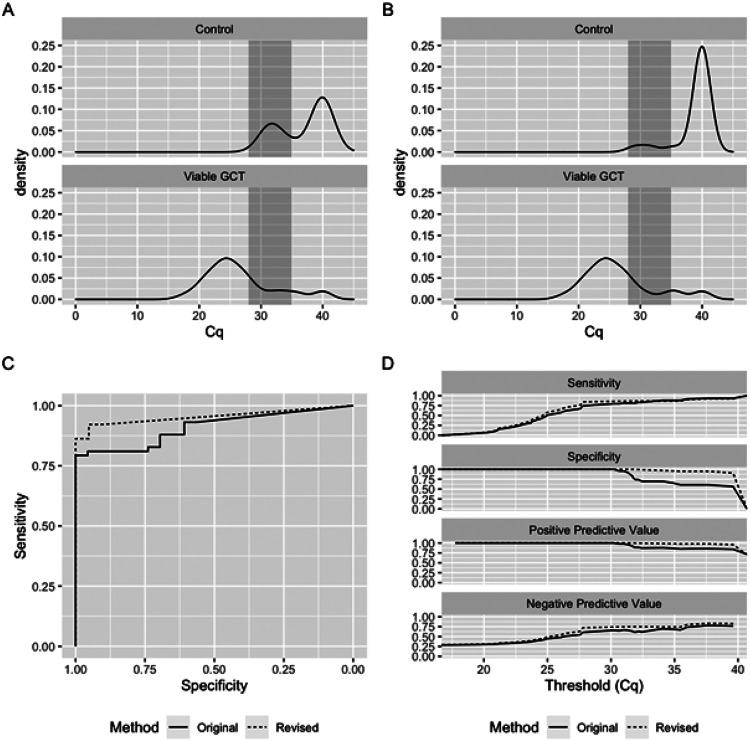
Establishment of indeterminate range in the serum miR-371a-3p test. **A-B:** Density plots of serum miR-371a-3p Cq values (n = 81) under original (A) or revised (B) method. Shaded region corresponds to indeterminate range (Cq 28-35). **C:** ROC plot showing performance of original (solid line) and revised (dashed line) methodology. **D:** Comparison of sensitivity, specificity, positive predictive value, and negative predictive value across all measured Cq thresholds using original (solid line) and revised (dashed line) methodology.

Mitigation of this misclassification is critical prior to clinical implementation of the test. We reasoned that defining an ‘indeterminate’ range based on the first distribution and repeating the qPCR for any sample that fell into that range would reduce misclassification from ~ 25% to ~ 6% (0.25 x 0.25 = 0.0625). Based on our established assay pipeline, we defined the indeterminate range as Cq 28–35, which approximates the mean of the first Cq peak ± 2 SDs in the controls. We then interrogated our aggregated data again to simulate how application of this revised methodology might improve viable GCT classification. To simulate the original methodology, the first chronological run per sample was selected. To simulate our revised methodology, the first chronological run per sample was selected unless its result fell into the indeterminate range (28 < Cq < 35). If so, the second chronological run was selected. Any sample that remained indeterminate after the second run was classified ‘indeterminate’ and removed from performance calculations. With this model, the original method had 81 runs. In the revised method, nine samples (11.1%) had two indeterminate results and were classified as truly indeterminate, leaving 72 runs. Two of these nine samples were in the Control group, and the remaining seven were in the Viable GCT group. We then compared the resulting Cq distributions, Fig. 2A-B. Application of the revised methodology prevented six false positives with accuracy improved from 0.85 to 0.93, and AUC from 0.909 to 0.954, Fig. 2C-D and **Supplementary Table 2**. False positives in the Control group declined from 8/23 (34.8%) to 2/23 (8.7%), supporting the observation that this event is stochastic in nature.

Application of revised methodology to an updated primary RPLND dataset

Improved performance of the serum miR-371a-3p test would allow for both early detection of recurrence and avoidance of unnecessary treatment. The detection of minimal residual disease (MRD) therefore carries great clinical significance in this context. As serum miR-371a-3p Cq is correlated with tumor burden, detection of MRD demands the greatest performance of this test. We therefore expanded a cohort of chemotherapy naïve patients receiving primary RPLND and compared the performance of the original and revised methodology.

Patient characteristics are summarized in Table 2. Thirty-two patients receiving primary RPLND were included in the present analysis. Most patients were clinical stage (CS) II (62.5%); 37.5% were CSI. At RPLND, nine patients (28.1%) had no viable tumor, 12 patients (37.5%) had pure seminoma, and 11 patients (34.4%) had non-seminomatous GCT. Pathologic stage (PS) was PS I in 28.1% and PS II in 71.9%.

**Figure 3:**
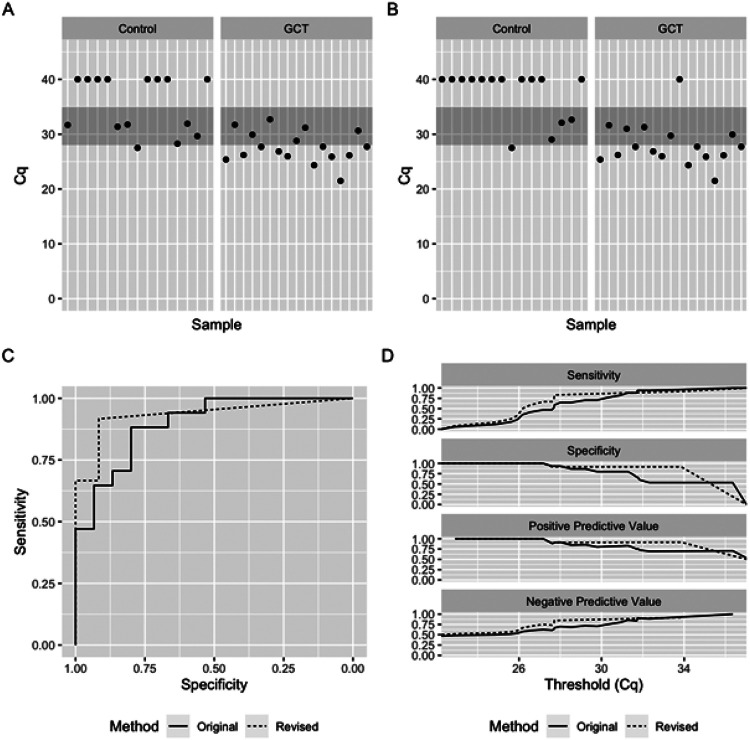
Revised method improves performance of serum miR-371a-3p test in patients with minimal residual disease. **A-B:** Cq values of serum miR-371a-3p using original (A) and revised (B) methodology. **C:** ROC plot showing performance of original (solid line) and revised (dashed line) methodology. **D:** Comparison of sensitivity, specificity, positive predictive value, and negative predictive value across all measured Cq thresholds using original (solid line) and revised (dashed line) methodology.

The median Cq for the Control group was 40 under the original and revised methodology. Median Cq for the Viable GCT group shifted from 27.7 under the original methodology to 26.2 under the revised methodology. After applying the revised method, eight samples remained truly indeterminate, which were removed from further analysis, Fig. 3A-B. Three of these samples were in the Control group, all of which harbored pure teratoma. The remaining five indeterminate samples were in the Viable GCT group. The AUC was 0.898 (95% CI: 0.79-1.00) with the original method and 0.934 (95% CI: 0.84-1.00) with the revised method, Fig. 3C. Application of the revised methodology improved most other metrics, including specificity (0.80 to 0.92) and PPV (0.83 to 0.92), Fig. 3D and **Supplementary Table 3**.

## Discussion

We report the use of raw circulating miR-371a-3p Cq values, instead of normalized data, for optimal assay performance with excellent interlaboratory concordance. qPCR assays are extensively and routinely used in clinical laboratories and often report results using raw Cq. Introduction of a normalization procedure increases costs and hampers translation into routine clinical testing. Due to the very high sensitivity of the circulating miRNA assay for viable GCT, we believed that additional normalization would be necessary to control for variation between runs. However, results from identical samples run in two independent laboratories suggest normalization may be harmful. The addition of these normalization procedures introduces additional technical variation due to the discordance of reference genes (cel-miR-39-3p and miR-30b-5p) without performance benefits.

Other groups used raw data in their assessments and retained high performance [15, 16]. However, assays used by these groups differ materially (e.g., the use of plasma extracts, detection by droplet digital PCR (ddPCR), and/or no pre-amplification). Since the largest miRNA studies to date, including a commercially available assay (miRdetect), were conducted with a serum qPCR-based method with pre-amplification, we felt it important to replicate these studies using this particular methodology.

**Figure 4:**
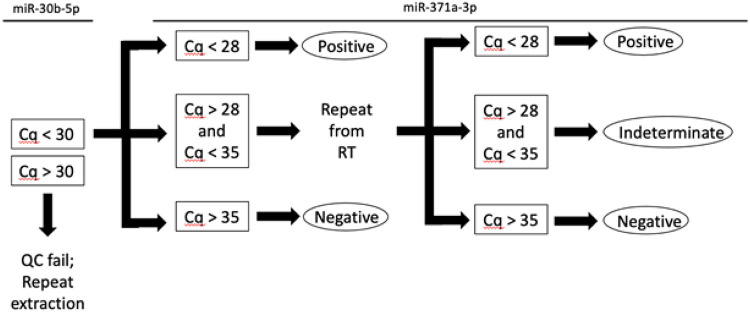
Decision-making flowchart for revised serum miR-371a-3p method. First, results of miR-30b-5p are evaluated for quality control. If Cq > 30, insufficient RNAs may have been isolated from the serum sample, and this sample should be reextracted. If Cq < 30, proceed to evaluation of miR-371a-3p. If Cq < 28, accept positive result. If Cq > 35, accept negative result. If 28 < Cq < 35, assay should be repeated from reverse transcription (RT) step. If Cq < 28 or Cq > 35, accept results as above. If 28 < Cq < 35 again, report indeterminate and recommend short interval follow up.

Critically, we have identified and established an indeterminate range to maintain assay performance of the circulating miR-371a-3p test. This arises from the observation in three separate laboratories that any given negative sample has an approximately 25% random or stochastic chance to return a spurious positive result. The existence of this reproducibility issue is further supported by an independent study reporting the existence of an indeterminate range in normalized values [17]. Additionally, Christiansen et al recently reported that the inclusion of the pre-amplification step improved sensitivity but also led to more false positives [18]. Dropping the assay cutoff below the first distribution would lead to an unacceptable drop in sensitivity. Instead, we elect to define an indeterminate range and rerun any indeterminate extract (Fig. 4). We have observed that upon repeat, most true positive samples will maintain a Cq value very close to the first run, while most true negative samples will yield a negative result. Because outcomes for viable GCT tend to be positive even in the case of recurrence, we recommend classification of any sample that returns an indeterminate result twice as a true indeterminate. In this clinical scenario, there is comparatively greater patient cost to over-treat than undertreat. Application of our revised method to an expanded cohort of patients with MRD improved specificity and PPV, demonstrating that these changes could prevent over-treatment.

Because many groups use a similar or identical protocol for this test, the question arises as to why this indeterminate range has not previously been described in detail. One contributing factor may be that larger retrospective non-blinded studies using this serum qPCR-based assays are focused on testicular GCT rather than retroperitoneal disease. Because circulating miR-371a-3p levels are dependent upon tumor burden, circulating miR-371a-3p is anticipated to be weakly positive in the context of MRD, rendering cutoff selection difficult. For example, the median Cq value for Viable GCT patients in our orchiectomy cohort [10] was 26.6, below the indeterminate range. However, the median Cq for our original primary RPLND cohort [9] was 29.3, within the indeterminate range. Additionally, a small number of spurious positive results in a control group may be written off as technical error and/or potential contamination, and the qPCR run repeated several times, subsequently yielding negative results. This enforces the utility of blinding technicians and analysts when conducting assays.

## Conclusion

We recommend three important modifications to serum miR-371a-3p assay protocols going forwards: 1) revise the test by applying cutoffs to raw Cq values instead of normalized values; 2) include endogenous (eg, miR-30b-5p) and exogenous (eg, cel-miR-39-3p) controls for quality control purposes; 3) include an indeterminate range to enhance specificity. These changes reduce the complexity and cost of the test while improving performance, particularly with regards to the detection of MRD. We believe the present work regarding reproducibility and thresholding provides a substantial step towards the clinical implementation of the serum miR-371a-3p assay for management of patients with viable GCT disease.

## Figures and Tables

**Table 1:** Performance metrics of raw and normalized values of serum miR-371a-3p test.

Orchiectomy (n = 69)	Cq	ΔCq	RQ
Threshold	32.75	18.03	23.46
AUC	0.98	0.98	0.98
Sensitivity	0.93	0.91	0.93
Specificity	1.00	1.00	1.00
1° RPLND (n = 24)	Cq	ΔCq	RQ
Threshold	36.35	21.92	20.59
AUC	0.97	0.97	0.97
Sensitivity	1.00	1.00	0.92
Specificity	1.00	0.92	0.92

**Table 2:** Patient characteristics of minimal residual disease dataset.

Age	Years	Median (IQR)	28 (23.5–35.0)
Race/Ethnicity	White Non-Hispanic	N (%)	18 (56.3)
	Hispanic		13 (40.6)
	Other		1 (3.1)
Body Mass Index	kg/m^2^	Median (IQR)	27.4 (23.7–29.8)
Primary Tumor Size	cm	Median (IQR)	3.3 (2.2-6.0)
Primary Histopathology	Seminoma	N (%)	9 (28.1)
	Non-Seminoma		21 (65.6)
	Burnt-Out Primary		2 (6.3)
Primary Tumor Lymphovascular Invasion (LVI)	No	N (%)	21 (65.6)
	Yes		11 (34.4)
Primary Tumor Rete Testis Invasion (RTI)	No	N (%)	20 (62.5)
	Yes		12 (37.5)
pT Stage	pT0	N (%)	2 (6.3)
	PT1		14 (43.8)
	pT2		16 (50.0)
cN Stage	cN0	N (%)	12 (37.5)
	cN1		15 (46.9)
	cN2		4 (12.5)
	cN3		1 (3.1)
S Stage	S0	N (%)	23 (71.9)
	S1		9 (28.1)
Clinical Stage (CS)	CS I	N (%)	12 (37.5)
	CS II		20 (62.5)
RPLND Histopathology	Benign	N (%)	9 (28.1)
	Seminoma		12 (37.5)
	Non-Seminoma		11 (34.4)
pN Stage	pN0	N (%)	9 (28.1)
	pN1		11 (34.4)
	pN2		11 (34.4)
	pN3		1 (3.1)
Pathologic Stage (PS)	PS I	N (%)	9 (28.1)
	PS II		23 (71.9)

## Data Availability

The data analyzed for this publication are available upon reasonable request from the corresponding author.
